# Carbon ion therapy for advanced sinonasal malignancies: feasibility and acute toxicity

**DOI:** 10.1186/1748-717X-6-30

**Published:** 2011-04-05

**Authors:** Alexandra D Jensen, Anna V Nikoghosyan, Swantje Ecker, Malte Ellerbrock, Jürgen Debus, Marc W Münter

**Affiliations:** 1 Dept of Radiation Oncology INF 400 69120 Heidelberg, Germany; 2 Dept. of Medical Physics Heidelberg Ion Therapy Centre (HIT) INF 450 69120 Heidelberg, Germany

## Abstract

**Purpose:**

To evaluate feasibility and toxicity of carbon ion therapy for treatment of sinonasal malignancies. First site of treatment failure in malignant tumours of the paranasal sinuses and nasal cavity is mostly in-field, local control hence calls for dose escalation which has so far been hampered by accompanying acute and late toxicity. Raster-scanned carbon ion therapy offers the advantage of sharp dose gradients promising increased dose application without increase of side-effects.

**Methods:**

Twenty-nine patients with various sinonasal malignancies were treated from 11/2009 to 08/2010. Accompanying toxicity was evaluated according to CTCAE v.4.0. Tumor response was assessed according to RECIST.

**Results:**

Seventeen patients received treatment as definitive RT, 9 for local relapse, 2 for re-irradiation. All patients had T4 tumours (median CTV1 129.5 cc, CTV2 395.8 cc), mostly originating from the maxillary sinus. Median dose was 73 GyE mostly in mixed beam technique as IMRT plus carbon ion boost. Median follow- up was 5.1 months [range: 2.4 - 10.1 months]. There were 7 cases with grade 3 toxicity (mucositis, dysphagia) but no other higher grade acute reactions; 6 patients developed grade 2 conjunctivits, no case of early visual impairment. Apart from alterations of taste, all symptoms had resolved at 8 weeks post RT. Overall radiological response rate was 50% (CR and PR).

**Conclusion:**

Carbon ion therapy is feasible; despite high doses, acute reactions were not increased and generally resolved within 8 weeks post radiotherapy. Treatment response is encouraging though follow-up is too short to estimate control rates or evaluate potential late effects. Controlled trials are warranted.

## Background

Sinonasal malignancies include malignant tumours of various histologies in the nasal cavity and paranasal sinuses. Squamous cell carcinomas account for the majority of these tumours [1-3], however, also various rare histologies such as adenoidcystic carcinoma, aesthesioneuroblastoma, and mucosal melanoma are found.

Due to limited accessibility of these sites and late occurrence of symptoms, patients are mostly diagnosed with advanced disease [4-6]. Traditionally, surgery has been the primary treatment modality for this disease. Faced with predominantly advanced tumour stages surgical resection is limited by the proximity of various critical structures such as eye and optic pathways. Extensive surgery in advanced sinonasal tumours can be very mutilating; in view of patients' quality of life, radicality of surgical resection can therefore rarely be achieved. In addition, mortality and complication rates are not insignificant [7] and substantially increasing with patient age [8].

Local relapse rates following surgical treatment of 50 - 60% [4,9] are consequently high; in high-risk situations such as involved or close surgical margins and advanced tumour stage, adjuvant radiotherapy is recommended [9-11].

In conventional treatment techniques, sufficient dose application in radiation therapy has been limited by dose to surrounding organs at risk and subsequent early and late toxicity leading to loss of vision in approximately one third of patients [12,13]. With the advent of more sophisticated radiation treatment techniques like 3D-conformal RT [14,15], intensity-modulated RT (IMRT) [16-19], and image-guided RT (IGRT) over the years, toxicities were effectively reduced while local control remained more or less stable [10,20]. First and predominant site of treatment failure in paranasal sinus and nasal cavity cancer remains in-field [21,22]. Multivariate analyses found local control strongly dependent on applied dose stressing the need for further dose escalation in RT [22]. Particle therapy was shown to improve local control in relatively radioresistant cancers of the head and neck [23,24], while increased biological efficiency (RBE) and physical properties of dose distributions with extremely sharp gradients also argue strongly in favour of this treatment [25-28]. With the permanent availability of particle therapy by the establishment of hospital-based sites, this treatment becomes more frequently though not commonly available. We would like to report initial outcome of carbon ion therapy of sinonasal malignancies with respect to acute toxicity and initial response at our facility.

## Methods

### Patients

29 patients with histologically proven or incompletely resected malignant tumors of the paranasal sinuses and nasal cavity were treated mostly with a combination of IMRT and carbon ion therapy or carbon ion therapy alone from November 2009 to August 2010. Prior RT was not an exclusion criterion if another course of radiation therapy was justifiable. Toxicity was assessed at completion of combination treatment and on each follow-up visit. Treatment response was evaluated according to RECIST based on contrast-enhanced MRI-scans at the first follow-up visit. Treatment-related toxicity was prospectively collected and patient data was retrospectively analysed.

### Radiotherapy

#### Immobilization/planning examinations

Patients were immobilized using individual scotch cast or thermoplastic head masks with shoulder fixation. Planning examinations consisted of a planning CT scan (3 mm slice thickness) with the patient positioned in the individual fixation device and contrast-enhanced MRI for 3D image correlation as a standard.

#### Target volumes/dose prescription

CTV1 (carbon ion boost) includes the macroscopic tumor/prior tumor bed with special focus on the R2/R1-area. In malignant salivary gland tumors, neural pathways to the base of skull (cave: perineural invasion and skip lesions) are also included in the CTV1. PTV1 consists of a 3 mm margin around the CTV1 but does not extend into critical organs at risk (i.e. brain stem, spinal cord).

We prescribe a dose of 24 GyE carbon ions in 3 GyE/fraction (5 fractions per week) to the CTV1, we aim at covering the CTV1 with the 95% prescription isodose. The carbon ion boost is given at the HIT (Heidelberg ion beam therapy centre).

CTV2 includes CTV1 with safety margins along typical pathways of spread. Only ipsilateral nodal levels (II and III) are included, however, in case the primary tumor is/was located at midline or crossing midline, bilateral nodal levels II and III are covered. In case there is pathological lymph node involvement, additional nodal levels are covered as indicated. CTV2 also encompasses the complete surgical operational area and takes account for set-up variations, hence corresponds to the PTV2 (CTV2 = PTV2).

50 Gy IMRT (inversely planned step-and-shoot or tomotherapy technique) in 25 fractions (5 fractions per week) are prescribed to the CTV2 (coverage at least with the 90% prescription isodose) taking into account doses applied by daily image guidance with MV-cone-beam CT. If necessary, daily pretreatment online correction of translational vectors was carried out.

In case of patients undergoing a second course of radiation, CTV1 includes the visible tumor only. Doses are prescribed individually depending on prior RT and interval between the two treatments. No elective nodal irradiation was performed in patients receiving carbon ions only.

### Particle therapy

The carbon ion therapy is given at the HIT after inverse treatment planning in active beam application (raster-scanning method) [29] with a horizontal, fixed beamline at 5 fractions per week (Tue - Sat).

A monoenergetic carbon ion beam with a full-width/half-maximum (FWHM) of 5 mm is extracted from the accelerator system (synchrotron) and magnetically deflected to subsequently scan all planned iso-energetic slices roughly corresponding to the tumor's radiological depth. Using this method, almost any desired dose distribution can be created and dose to surrounding critical structures can be minimized.

Inverse treatment planning was carried out on a dedicated Siemens treatment planning system (TPS^®^). As ion beams exhibit an increased biological effective dose depending on various factors, these need to be included within the planning algorithm. Therefore, TPS^® ^additionally offers methods for inverse treatment planning and biological RT treatment optimization for particle therapy. In addition, steering parameters for scanned ion beams need also be calculated by the TPS.

Daily image guidance consisted of orthogonal x-ray controls in treatment position with the x-ray tube/receptor mounted on a robot to allow imaging in almost any treatment table position. After acquisition of orthogonal x-rays, an automatic 2D-3D pre-match was carried out (Siemens syngo PT treatment) and verified by the radiotherapist/radiation oncologist with regard to bony anatomy. Manual adjustment of the match was carried out on-line and the resulting correction vector, including rotations, subsequently applied to the patient position. Patient position was controlled in each session and shifts were always corrected using a robotic table allowing position correction in six degrees of freedom.

### Intensity-modulated radiotherapy (IMRT)

IMRT is carried out at a 6 MV linear accelerator after inverse treatment planning either in step-and-shoot technique or as tomotherapy at 5 fractions per week (Mon - Fri). Image guidance consists of regular MV-cone-beam CTs with online correction prior to treatment application in 3 degrees of freedom. Doses delivered by the MV-imaging were taken into account for the total applied dose.

### Radiotherapy plan evaluation/dose constraints

IMRT and carbon ion treatment plans had to be optimized and evaluated separately according to the following criteria: <20% of the CTV1 should receive ≥110% of the prescribed dose, <5% of CTV1 or CTV2 should receive ≤90% of the prescribed dose, and <2% or 2 cc of tissue outside the CTVs should receive ≥110% of the prescribed dose to the CTV1. In addition, the following normal tissue constraints served as a basis for individual plan evaluation. These constraints were applicable for the summation (carbon ion and photon IMRT) plan at standard fractionation (2 Gy/fraction).

• Spinal cord: the dose to any point within the spinal cord should not exceed 5045 Gy to any volume larger than 0.03 cc.

• Brain stem: the tolerated dose is 54 Gy; maximum tolerated dose in volumes of ≤1cc: 60 Gy.

• Optic chiasm/optic nerves: maximum dose to these structures should be ≤54 Gy, in case this dose limit cannot be kept without compromising target volume coverage, these issues were discussed with the patient and decisions made accordingly.

• Eyes: maximum doses ≤45 Gy to the posterior bulb/retina; doses to the whole eye were reduced as low as reasonably achievable without compromising target volume coverage

• Parotid glands: mean dose to at least one gland below 26 Gy; alternatively at least 20 cc of the combined volume of both parotid glands to <20 Gy or at least 50% of one gland to <30 Gy.

### Follow-up

First follow-up examination including clinical examination and diagnostic, contrast-enhanced MRI was carried out 6 weeks post completion of radiation treatment. Further controls including MRI were scheduled for 3, 6, and 12 months thereafter.

Patients were also encouraged to undergo regular check-ups incl. full ENT and ophthalmologic clinical examinations in regular intervals.

### Analysis

Evaluation of toxicity was carried out according to NCI CTCAE v. 4.0, treatment was evaluated using the RECIST-criteria [30] based on available follow-up scans (CT or MRI) and clinical examinations 6-8 weeks post completion of therapy.

## Results

Twenty-nine patients with sinonasal malignancies were treated from 11/2009 to 08/2010. Median age was 57 years [range: 20 - 77 years]. Median follow-up was 5.1 months [range: 2.4 - 10.8 months]. All patients were alive at last follow-up time. Fifty-nine percent (17 pts) received treatment as definitive radiation therapy either due to surgical inoperability or R2-resections, 9 patients were treated for locally recurrent disease; 2 patients received carbon ion therapy as a second course of radiation. Most tumours were located in the maxillary sinus, however due to extensive disease, the primary site could not be identified in 3 patients. Most of the patients had histologically proven adenoid cystic carcinoma or malignant melanoma, tumour stages were advanced (T4) in most of the cases. Four patients had undergone surgical orbital exenteration, 2 patients induction chemotherapy with no sign of persistent tumour in one patient. Another patient received radiation therapy as combined radioimmunotherapy with cetuximab weekly (table 1).

**Table 1:** patient baseline characteristics

**site**	maxillary sinus	19 pts
	nasal cavity	3 pts
	ethmoid sinus	3 pts
	sphenoid sinus	1 pt
	pansinus	3 pts
		
**histology**	adenoidcystic carcinoma	20 pts
	malignant melanoma	3 pts
	undifferentiated carcinoma	1 pt
	chordoma	1 pt
	chondrosarcoma	1 pt
	osteosarcoma	1 pt
	ameloblastic carcinoma	1 pt
	malignant peripheral nerve sheath tumour	1 pt
		
**stage**	T4	18 pts
	T3	4 pts
	T2	1 pt
	N+	1 pt
	not applicable	5 pts
		
**therapy**	primary	20 pts
	local relapse	9 pts
	reirradiation	2 pts
	R1-resected	11 pts
	R2-resected/definitive RT	17 pts
	orbital exenteration	4 pts
	post-induction	2 pts
	comb. Radioimmunotherapy	1 pt

**Figure 1:**
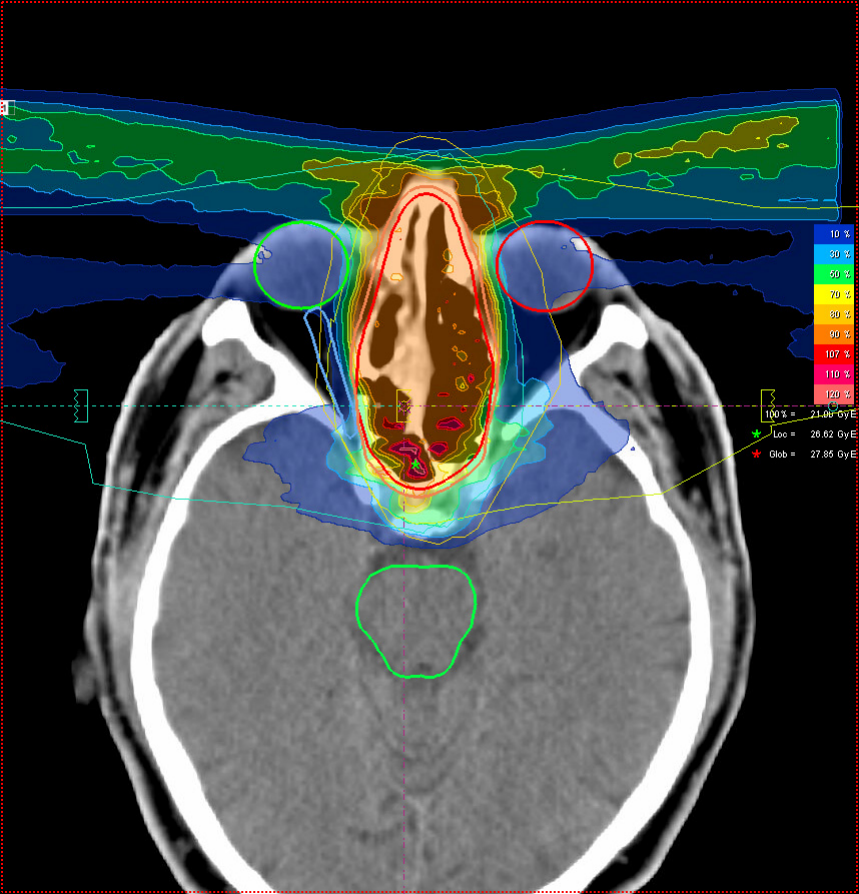
61 year-old patient with malignant melanoma pT4 cN2b: carbon ion 3-field IMPT (axial)

**Figure 2:**
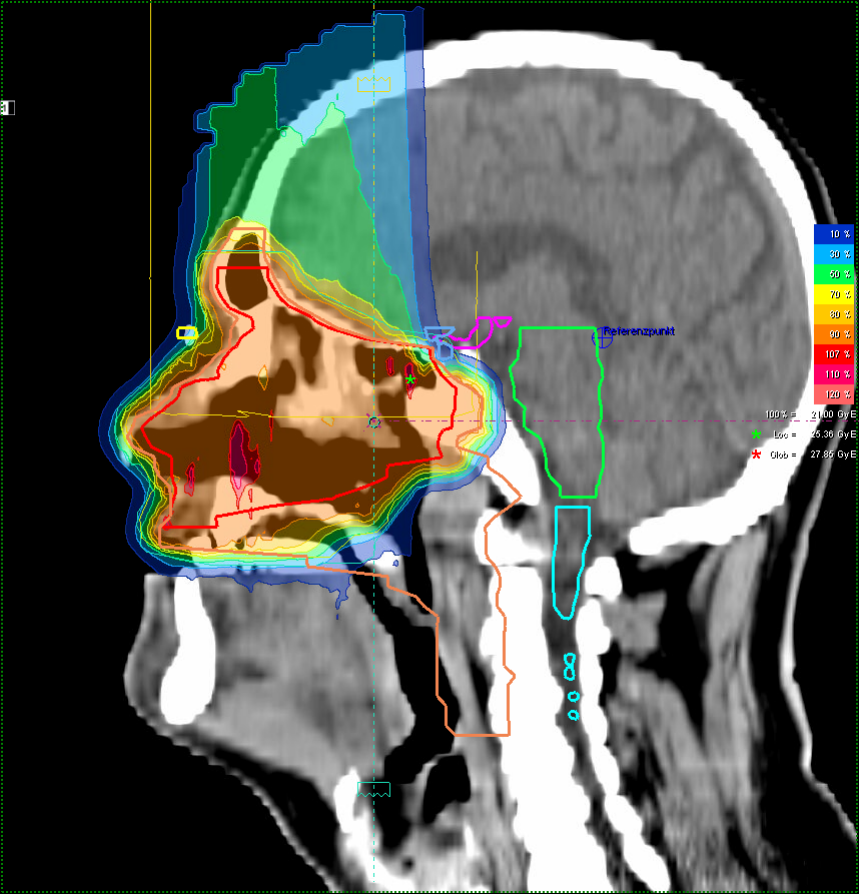
61 year-old patient with malignant melanoma pT4 cN2b: carbon ion 3-field IMPT (sagittal)

Most patients (25/29 pts) received mixed-beam radiotherapy consisting of IMRT either in step-and-shoot technique (22 pts) or tomotherapy (3 pts) and carbon ion boost. Four patients received carbon ion therapy only. Median total dose applied was 73 GyE [range: 70 - 75 GyE]. Treatment volumes were large with a median CTV1 volume of 129.5 cc and CTV2 volume of 395.8 cc (table 2). Carbon ion therapy was applied over 2 non-coplanar fields after inverse treatment planning using single-beam optimization (26 pts) and intensity-modulated particle therapy (IMPT) in 2 cases. Only one patient with pansinus tumour needed 3 fields. Treatment times including patient positioning and position verification were typically between 35 and 55 minutes per fraction compared to approx. 20 min for standard IMRT. Table 3 summarizes dose-volume statistics for respective critical structures. Figure 1, 2, and 3 show an exemplary carbon ion and IMRT (Figure 4, 5, and 6) treatment plan of a patient with adenoidcystic carcinoma.

**Table 2:** treatment characteristics; C12: = carbon ion therapy

	**median dose/GyE or Gy**	**range/GyE or Gy**
**C12**	24	21 - 60
**IMRT**	49	47 - 51
**total**	73	70 - 75
	**median volume/cc**	**range/cc**
**CTV1**	129.5	41.9 - 422.0
**CTV2**	395.8	100.2 - 1246.8
		
combined treatment	25 pts	(8 fractions C12)
step& shoot IMRT	22 pts	
tomotherapy	3 pts	
C12 only	4 pts	(15-20 fractions)

**Table 3:** treatment plan parameters; C12:= carbon ion therapy

		**max (Gy/GyE)**							
		**ipsilateral eye**	**contralateral eye**	**ipsilateral optic nerve**	**contralateral optic nerve**	**optic chiasm**	**brain stem**	**spinal cord**	**ipsilateral lens**
**C12**	median	21,5	11,5	17,3	13,6	8,3	14,1	0	7,4
	max	50,1	48,5	53,9	51,1	51,9	49,4	14,7	23,3
	min	2,3	2	0	0	0	0	0	0
**IMRT**	median	45,7	27,9	42,4	32,5	28,6	38,9	33,9	23,8
	max	53,3	45,5	50	51,8	45,5	46,3	40,7	41,1
	min	31,4	10,2	4,6	4,2	4,1	22,1	0	17,5
**summation**	median	63,6	35,7	56,2	43,1	31,5	47,5	30	22,3
	max	74	65,3	67	61,5	58,1	65,9	49,7	59,9
	min	18,6	0	4,6	0	0	0	0	4,6
									
									
		**max (Gy/GyE)**			**median (Gy/GyE)**				
		**contralateral lens**	**ipsilateral mandibular joint**	**contralateral mandibular joint**	**ipsilateral parotid**	**contralateral parotid**	**ipsilateral eye**	**contralateral eye**	
**C12**	median	8,2	20,7	1,1	2,1	0	5,3	2	
	max	40	25,9	23,5	23,2	24,9	22,6	31,2	
	min	0	6,5	0	0	0	0	0	
									
**IMRT**	median	12,7	49,6	8,3	23	8,7	25,4	15,5	
	max	30,1	51	53,1	32,5	27,2	36,1	27,1	
	min	10,7	24,7	4,7	1,7	1,3	3	2,2	
									
**summation**	median	15	31,2	7,5	21,3	8	21,1	16,9	
	max	40,8	75,5	76,6	47,6	48,5	46,3	38,7	
	min	2	7,5	0,2	0	0	0	0	

**Figure 3:**
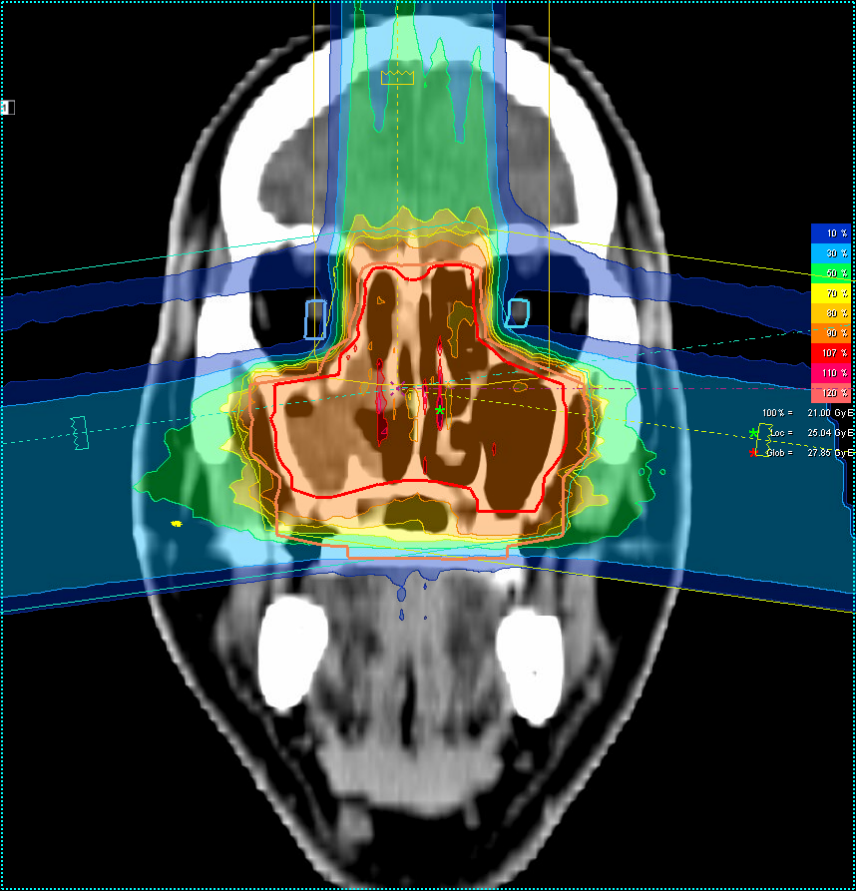
61 year-old patient with malignant melanoma pT4 cN2b: carbon ion 3-field IMPT (coronal)

**Figure 4:**
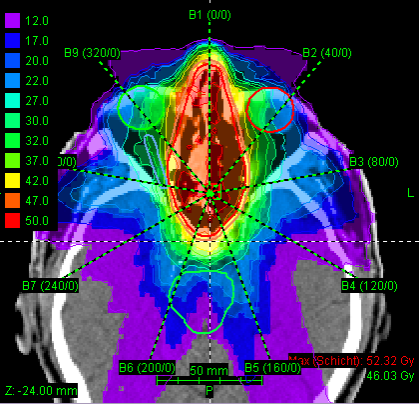
61 year-old patient with malignant melanoma pT4 cN2b: step-and-shoot IMRT plan using 9 coplanar beams, dose legend in Gy (axial)

**Figure 5:**
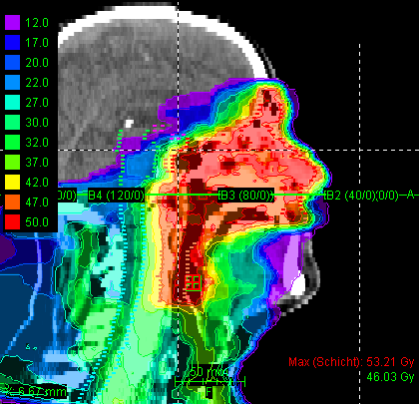
61 year-old patient with malignant melanoma pT4 cN2b: step-and-shoot IMRT plan using 9 coplanar beams, dose legend in Gy (sagittal)

**Figure 6:**
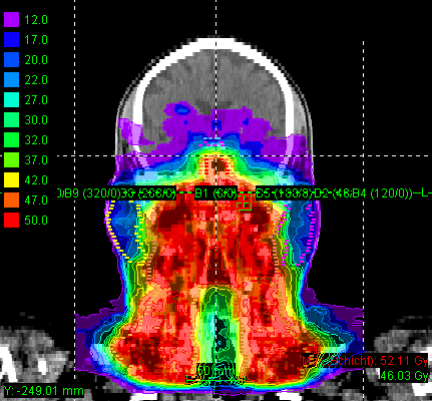
61 year-old patient with malignant melanoma pT4 cN2b: step-and-shoot IMRT plan using 9 coplanar beams, dose legend in Gy (coronal)

At first follow-up 6 weeks post completion of radiation therapy, two patients showed complete, 6 patients good partial remissions. Eight patients had stable disease, among them the patients with chordoma, chondrosarcoma, and osteosarcoma. Eleven of the postoperatively treated patients and the patient who had undergone induction chemotherapy for undifferentiated paranasal sinus carcinoma showed no signs of disease. One patient with malignant melanoma however developed a local recurrence within the high dose area (total dose 73.1 GyE) as well as distant metastases (liver, bone) at first follow-up, another patient also with malignant melanoma developed distant metastases four months after completion of radiotherapy.

Treatment was tolerated well with 7 cases of acute grade 3 toxicity (mucositis: 5 pts; dysphagia: 2 pts) at completion of radiotherapy. There were no treatment interruptions or any case of grade 4 or 5 acute toxicity. Most patients developed moderate mucositis, dermatitis, xerostomia, or dysgeusia leading to mild or moderate dysphagia. Two patients, both of them with extensive treatment fields, needed supportive therapy by parenteral nutrition or feeding tube. Due to the close proximity of the treatment fields, 6 patients developed radiation-induced conjunctivitis (table 4). Six to eight weeks (first follow-up) post treatment, only 3 patients showed grade 3 reactions (serous otitis) with a drainage tube in place. Many patients still complained of residual alterations in taste, however all of them described these symptoms as gradually resolving; 12 patients still had mild xerostomia and 2 patients presented with residual mucositis at their first follow-up. There were no cases of early visual impairment or residual conjunctivitis (table 5).

**Table 4:** toxicity at completion of RT

	**CTC grade**			
**toxicity**	**I (pts)**	**II (pts)**	**III (pts)**	
mucositis	9	14	5	
dermatitis	17	8	0	
desquamation	1	0	0	
dysphagia	8	5	2	
xerostomia	22	0	0	
otitis	0	3	0	
conjunctivitis	0	6	0	
serous otitis	0	2	0	(prae-therapeutic: 2 pts)
watering eyes	2	0	0	
xerophthalmia	1	0	0	
lymphoedema	3	0	0	
transitory change/loss of taste	12	10	0	
				
prophylactic feeding tube	3 pts			
weight loss	10 pts	2-8 kg		
praetherapeutic mandibular joint fibrosis	5 pts			

**Table 5:** toxicity at 8 weeks post completion of RT

	**CTC grade**			
**toxicity**	**I (pts)**	**II (pts)**	**III (pts)**	
mucositis	2	0	0	
dermatitis	0	0	0	
desquamation	0	0	0	
dysphagia	5	0	0	
xerostomia	12	0	0	
otitis	2	3	0	
hearing impairment	2	0	0	
conjunctivitis	0	0	0	
serous otitis	1	0	0	(prae-therapeutic: 2 pts)
watering eyes	0	0	0	
xerophthalmia	1	0	0	
lymphoedema	0	0	0	
transitory change/loss of taste	16	0	0	

## Discussion

Treatment for sinonasal malignancies remains a complex issue even in the days of modern surgical and radiotherapeutic techniques. Hence Chen and co-workers [20] were asking a very important question: Are we making progress?

Rates of in-field local recurrences call for further dose escalation within the treatment volume [21,22]. However, especially in tumours of the paranasal sinuses and nasal cavity, treatment-related toxicity has so far limited attempts of dose escalation. With the introduction of modern radiotherapy techniques, side-effects could be reduced while local control remained largely unchanged [20]. The clinical establishment of carbon ion therapy however, has seen the improvement of local control rates in adenoidcystic carcinoma [23,24,31,32] where in contrast to neutron therapy [33-36], no increased rates of toxicity were observed. Also, physical properties of heavy charged particles such as carbon ions as well as active scanning methods allow generation of highly conformal dose distributions and extremely steep dose gradients. Therefore, application of heavy ion therapy for the treatment of paranasal sinus tumours seemed obvious.

While it could be shown in planning comparisons that particle dose distributions are indeed superior to conventional and IMRT treatment plans [27,28,37], this still needs to be clinically demonstrated. With the more widespread availability of particle therapy in the near future - by 2012, there will be 5 centres offering particle therapy in Germany alone - treatment-related toxicity will be an important issue in the treatment of this disease. Since it will take years to evaluate treatment late effects, it is important to analyse and compare treatment-related acute reactions with past experience in the photon world at an early point in time as a predictor of late toxicity.

In the 29 patients treated in this series, no unexpected toxicity was seen: as reported by other groups [15,16,19,38,39], the vast majority of patients showed mucositis of some degree, a few groups did not observe any or only marginal grade 3 mucositis [15,19], however it needs to be emphasized that these reports included no [15] or less than 50% [19] T4 tumours, hence irradiation volumes will be smaller and consequently rates of higher grade acute mucositis will be lower. The rate of grade 3 toxicity in our patient cohort was 17.2% (5/29 pts) and ≥grade 2 65.5% (19/29 pts), which is in good agreement with results reported by Zenda et al (mucositis CTC grade 3: 21%) with a similar dose and target volume concept [40]. Though the rate of acute toxicity grade 2/3 in our patients is somewhat higher than reported by Wu et al [39], the authors did not describe their dose concept and it is unclear which total dose these patients received. Of course, occurrence of acute reactions is dependent on treated volume and absolute dose applied. Median doses reported by the various groups have been up to approximately 70 Gy [17-20,22,38]. Although postoperative radiation therapy could improve local control in sinonasal malignancies, first site of treatment failure still remains in-field, therefore further dose escalation above 65 GyE [18] is justified. In view of the median dose of 73 GyE and a median treatment volume (CTV2) of approximately 400 cc in our patient cohort the rate of mucositis observed is hardly surprising and within the published range. Although maximum doses to the ipsilateral eye were comparatively high [10,17,18], still we have only observed one case of xerophthalmia (CTC grade 1) and no ocular/visual toxicity so far. High maximum total doses to optic structures were caused by extensive tumours directly adjacent to the optic apparatus; however, due to steep gradients achieved by carbon ion and IMRT treatment, these doses were only received by small parts of the organ and median (total) doses were generally kept low (21.1 GyE ipsilateral and 16.9 GyE contralateral eye). Xerostomia and alterations of taste had also largely resolved 6-8 weeks post completion of radiotherapy. So far, there is no indication of lingering higher grade toxicity.

Unfortunately, there is very little data available for treatment of tumours in the paranasal sinuses or nasal cavity using either protons or heavier charged particles. Neutron therapy did yield comparatively good control rates, due to increased acute and late toxicity [34,41] as well as handling properties this treatment was abandoned in many places. Four groups have reported their results with particle therapy in this setting [32,38]. Mizoe et al evaluated two hypofractionated dose escalation regimens for advanced head and neck cancers of various histologies, among them squamous cell carcinomas, adenoidcystic carcinomas, and mucosal malignant melanomas. Nine of the thirty-six reported cases were located in the paranasal sinuses or nasal cavity. In their analysis, there were 7 cases of grade 3 skin and 1 case of grade 3 mucous membrane toxicity, however, toxicity was not analysed with regard to tumour site [32]. Also, this working group employed carbon ions only whereas we have mostly used a mixed beam regimen in order to account for potential locoregional tumour spread. One would of course already expect some degree of mucositis caused by the photon part of our treatment, therefore comparison of our results with carbon ion therapy only is difficult. In addition, HIMAC uses passive beam application: although efficiency of the beam is low requiring higher beam intensities than spot scanning methods, robustness of the system is high in view of potential positioning errors or anatomical changes (tissue swelling etc). Therefore systematic image guidance (i.e. pre-treatment position controls) needs to be implemented to maintain target coverage/normal tissue sparing and hence low toxicity profile in active beam application systems.

Zenda et al. recently published a pilot study using proton therapy to 60 GyE in 15 fractions as a nonsurgical treatment alternative reporting similar treatment-related acute toxicity as in our cohort with promising control rates [40]. Truong and co-workers treated patients with a combination of photon and proton therapy and observed a grade 3 mucositis rate of 30% and ≥grade 2 of 70% [38]. Apart from one patient who developed meningitis due to cerebrospinal fluid leak, these authors could not find any major late toxicity associated with their treatment at longer follow-up. Seven out of 36 patients developed acute radiation-related toxicity (conjunctivitis and epiphora) in the cohort published by Weber et al [42] treated with a combination of photon and proton RT. However, 13 out of the 36 patients developed late ocular complications; absolute doses to the GTV and optic structures were also seen as an important predictor of late radiation induced complications [42]. Though visual impairment most often develops after a longer interval post treatment - approximately 20 months in the cohort described by Hasegawa et al, it may be observed as early as 5 months post RT [43] with the latency period correlating to the dose to the optic structures [42].

In the available literature, there is also little data with regard to treatment response. In patients with visible residual tumour, we have observed an overall response rate of 7/17 patients (41.2%) including patients with chordoma and osteosarcoma, where fast tumour shrinkage is generally not expected. Complete responses were seen in a patient with large adenoidcystic carcinoma and ameloblastic carcinoma of the maxillary sinuses. One patient with R1-resected malignant melanoma however did develop an in-field recurrence in addition to distant disease progression; another patient with malignant melanoma also showed distant failure but stayed locally controlled. Overall treatment response after carbon ion therapy only was reported at 80,6% in the Japanese series [32], again, this is given for the whole patient cohort, analysis of the subset of tumours in the paranasal sinuses/nasal cavity is not available. As mentioned before, in this group consisted of more patients with squamous cell carcinoma and malignant melanoma [32]. Local tumour control at the time of evaluation was achieved in all but one patient (96,6%). However, due to short follow-up in our series, estimates of local control and comparison with results achieved by other groups (up to 86% at 2 years [14,15,17,18] and up to 74% at 5 years [18-21]) are not possible. All in all our tumour control so far seems encouraging though further follow-up is definitely needed to support initial results.

Our patient cohort mainly consisted of patients with adenoidcystic carcinoma of the paranasal sinuses and only to a small part of malignant mucosal melanoma and other rare histologies. This cohort does probably not reflect overall incidence of tumour entities in these sites, where squamous cell carcinoma and to a lesser extent malignant melanoma would be expected to be more frequent [1,9]. However, our main objective was to investigate accompanying early toxicity of our treatment for irradiation in this area of the body, therefore actual histologies are not as relevant. Another limitation to this analysis is, of course, comparatively short follow-up of our patients and no conclusion regarding potential late effects can be drawn yet. In view of the fact this treatment is comparatively new and will be more commonly available in the future, we still think potential side effects need early attention to prevent a large number of patients being treated before evaluation might reveal higher toxicity rates.

So radiotherapy for sinonasal cancers has dramatically improved within the past decade: treatment-related side-effects could be reduced by the introduction of new and sophisticated treatment techniques. Faced with sometimes unsatisfactory local control in this disease though, there is room still for improvement, which will also be based on dose escalation.

The best way to evaluate the risk benefit ratio of this treatment though is treatment of this indication within clinical trials. Hence, a phase II trial evaluating acute and late toxicity of combined IMRT and carbon ion boost for this indication is currently under way and will open for patient accrual by the end of 2010.

## Conclusion

Despite high delivered dose, this therapy is feasible, acute reactions were not increased as compared to 3D and IMRT treatment techniques and generally resolved within 6-8 weeks post radiotherapy. Treatment response is encouraging though follow-up is too short to estimate control rates or evaluate potential late effects. Controlled trials are needed to investigate these issues in a controlled setting.

## Competing interests

The authors declare that they have no competing interests.

## Authors' contributions

ADJ, AVN, MWM were responsible for treatment concepts and patient care, SE, ME for technical treatment planning and quality control, and JD and MWM for conceptual design. All authors read and approved the final manuscript.

## References

[B1] Muir CS Nectoux J Descriptive epidemiology of malignant neoplasms of nose, nasal cavities, middle ear and accessory sinuses Clin Otolaryngol Allied Sci 1980 5 195 211 10.1111/j.1365-2273.1980.tb01647.x 6996871

[B2] Rousch G Epidemiology of cancer of nose and paranasal sinus carcinomas: current concepts Head Neck Surg 1979 2 3 11 10.1002/hed.2890020103 400658

[B3] Tufano RP Mokadam NA Montone KT Weinstein GS Chalian AA Wolf PF Weber RS Malignant tumors of the nose and paranasal sinuses: hospital of the University of Pennsylvania experience 1990 - 1997 Am J Rhinol 1999 13 117 123 10.2500/105065899782106698 10219440

[B4] Cantu G Bimbi G Miceli R Mariani L Colombo S Riccio S Squadrelli M Battisti A Pompilio M Rossi M Lymph node metastases in malignant tumors of the paranasal sinuses: prognostic value and treatment Arch Otolaryngol Head Neck Surg 2008 134 170 177 10.1001/archoto.2007.30 18283160

[B5] Harbo G Grau C Bundgaard T Overgaard M Elbrond O Sogaard H Overgaard J Cancer of the nasal cavity and paranasal sinuses. A clinico-pathological study of 227 patients Acta Oncol 1997 36 45 50 10.3109/02841869709100731 9090965

[B6] Myers LL Nussenbaum B Bradford CR Teknos TN Esclamado RM Wolf GT Paranasal sinus malignancies: an 18-year single institution experience Laryngoscope 2002 112 1964 1969 10.1097/00005537-200211000-00010 12439163

[B7] Ganly I Patel SG Singh B Kraus DH Bridger PG Cantu G Cheesman A De Sa G Donald P Fliss D Gullane P Janecka I Kamata SE Kowalski LP Levine P Medina LR Pradhan S Schramm V Snyderman C Wei WI Shah JP Complications of craniofacial resection for malignant tumors of the skull base: report of an International Collaborative Study Head Neck 2005 27 445 451 10.1002/hed.20166 15825205

[B8] Ganly I Gross ND Patel SG Bilsky MH Shah JP Kraus DH Outcome of craniofacial resection in patients 70 years of age and older Head Neck 2007 29 89 94 10.1002/hed.20487 16983689

[B9] Genden EM Okay D Stepp MT Rezaee RP Mojica JS Buchbinder D Urken ML Comparison of functional and quality-of-life outcomes in patients with and without palatomaxillary reconstruction: a preliminary report Arch Otolaryngol Head Neck Surg 2003 129 775 780 10.1001/archotol.129.7.775 12874081

[B10] Hoppe BS Stegman LD Zelefsky MJ Rosenzweig KE Wolden SL Patel SG Shah JP Kraus DH Lee NY Treatment of nasal cavity and paranasal sinus cancer with modern radiotherapy techniques in the postoperative setting - the MSKCC experience Int J Radiat Oncol Biol Phys 2007 691 702 17161557 10.1016/j.ijrobp.2006.09.023

[B11] Katz TS Mendenhall WM Morris CG Amdur RJ Hinerman RW Villaret DB Malignant tumors of the nasal cavity and paranasal sinuses Head Neck 2002 24 821 829 10.1002/hed.10143 12211046

[B12] Parsons JT Mendenhall WM Mancuso AA Cassisi NJ Million RR Malignant tumors of the nasal cavity and ethmoid and sphenoid sinuses Int J Radiat Oncol Biol Phys 1988 14 11 22 10.1016/0360-3016(88)90044-2 3335447

[B13] Shukovsky LJ Fletcher GH Retinal and optic nerve complications in a high dose irradiation technique of ethmoid sinus and nasal cavity Radiology 1972 104 629 634 4626287 10.1148/104.3.629

[B14] Snyers A Janssens GORJ Twickler MB Hermus AR Takes RP Kappelle AC Merkx MA Dirix P Kaanders JH Malignant tumors of the nasal cavity and paranasal sinuses: long-term outcome and morbidity with emphasis on hypothalamic-pituary deficiency Int J Radiat Oncol Biol Phys 2009 73 1343 1351 10.1016/j.ijrobp.2008.07.040 18963535

[B15] Dirix P Nuyts S Geussens Y Jorissen M Vander Poorten V Fossion E Hermans R Van den Bogaert W Malignancies of the nasal cavity and paranasal sinuses: long-term outcome with conventional or three-dimensional conformal radiotherapy Int J Radiat Oncol Biol Phys 2007 69 1042 1050 10.1016/j.ijrobp.2007.04.044 17570610

[B16] Dirix P Vanstraelen B Jorissen M Vander Poorten V Nuyts S Intensity-modulated radiotherapy for sinunasal cancer: improved outcome compared to conventional radiotherapy Int J Radiat Oncol Biol Phys 2010 in press 10.1016/j.ijrobp.2009.09.067 20338694

[B17] Daly ME Chen AM Bucci MK El-Sayed I Xia P Kaplan MJ Eisele DW Intensity-modulated radiation therapy for malignancies of the nasal cavity and paranasal sinuses Int J Radiat Oncol Biol Phys 2007 67 151 157 10.1016/j.ijrobp.2006.07.1389 17189068

[B18] Hoppe BS Wolden SL Zelefsky MJ Mechalakos JG Shah JP Kraus DH Lee N Postoperative intensity-modulated radiation therapy for cancers of the paranasal sinuses, nasal cavity, and lacrimal glands: technique, early outcome, and toxicity Head Neck 2008 30 925 932 10.1002/hed.20800 18302261

[B19] Madani I Bonte K Vkaet L Boterberg T De Neve W Intensity-modulated radiotherapy for sinonasal tumors: Ghent University Hospital update Int J Radiat Oncol Biol Phys 2009 73 424 432 10.1016/j.ijrobp.2008.04.037 18755554

[B20] Chen AM Daly ME Bucci MK Xia P Akazawa C Quivey JM Weinberg V Garcia J Lee NY Kaplan MJ El-Sayed I Eisele DW Fu KK Phillips TL Carcinomas of the paranasal sinuses and nasal cavity treated with radiotherapy at a single institution over five decades: are we making improvement? Int J Radiat Oncol Biol Phys 2007 69 141 147 10.1016/j.ijrobp.2007.02.031 17459609

[B21] Chen AM Daly ME El-Sayed I Garcia J Lee NY Bucci MK Kaplan MK Patterns of failure after combined-modality approaches incorporating radiotherapy for sinonasal undifferentiated carcinoma of the head and neck Int J Radiat Oncol Biol Phys 2008 70 338 343 10.1016/j.ijrobp.2007.06.057 18207030

[B22] Hoppe BS Nelson CJ Gomez DR Stegman LD Wu Aj Wolden SL Pfister DG Zelefsky MJ Shah JP Kraus DH Lee NY Unresectable carcinoma of the paranasal sinuses: outcome and toxicities Int J Radiat Oncol Biol Phys 2008 72 763 769 10.1016/j.ijrobp.2008.01.038 18395361

[B23] Schulz-Ertner D Nikoghosyan A Didinger B Munter M Jäkel O Karger CP Debus J Therapy strategies for locally advanced adenoid cystic carcinomas using modern radiation therapy techniques Cancer 2005 104 2 338 44 10.1002/cncr.21158 15937907

[B24] Schulz-Ertner D Nikoghosyan A Jäkel O Haberer T Kraft G Scholz M Wannenmacher M Debus J Feasibility and toxicity of combined photon and carbon ion radiotherapy for locally advanced adenoid cystic carcinomas Int J Radiat Oncol Biol Phys 2003 56 391 398 10.1016/S0360-3016(02)04511-X 12738314

[B25] Brizel DM Light K Zhou SM Marks LB Conformal radiation therapy treatment planning reduces the dose to the optic structures for patients with tumors of the paranasal sinuses Radiother Oncol 1999 51 215 218 10.1016/S0167-8140(99)00043-2 10435816

[B26] Huang D Xia P Akazawa P Quivey JM Verhey LJ Kaplan M Lee N Comparison of treatment plans using intensity-modulated radiotherapy and three-dimensional conformal radiotherapy for paranasal sinus carcinoma Int J Radiat Oncol Biol Phys 2003 56 158 168 10.1016/S0360-3016(03)00080-4 12694834

[B27] Chera BS Malyapa R Louis D Mendenhall WM Li Z Lanza DC Yeung D Mendenhall NP Proton therapy for maxillary sinus carcinoma Am J Clin Oncol 2009 32 296 303 10.1097/COC.0b013e318187132a 19433966

[B28] Lomax AJ Goitein M Adams J Intensity modulation in radiotherapy: photons versus protons in the paranasal sinus Radiother Oncol 2003 66 11 18 10.1016/S0167-8140(02)00308-0 12559516

[B29] Haberer T Becher W Schardt D Kraft G Magnetic scanning system for heavy ion therapy Nucl Instr Meth Phys Res 1993 330 296 305 10.1016/0168-9002(93)91335-K

[B30] Therasse P Arbuck SG Eisenhauer EA Wanders J Kaplan RA Rubinstein L Verweij J Van Glabbeke M van Oosterom AT Christian MC Gwyther SG New guidelines to evaluate the response to treatment in solid tumors J Natl Cancer Inst 2000 92 205 216 10.1093/jnci/92.3.205 10655437

[B31] Huber PE Debus J Latz D Zierhut D Bischof M Wannenmacher M Engenhart-Cabillic R Radiotherapy for advanced adenoid cystic carcinoma: neutrons, photons or mixed beam? Radiother Oncol 2001 59 2 161 7 10.1016/S0167-8140(00)00273-5 11325445

[B32] Mizoe JE Tsujii H Kamada T Matuoka Y Tsuji H Osaka Y Hasegawa A Yamamoto N Ebihara S Konno A Organizing Committee for the Working Group for Head-And-Neck Cancer. Dose escalation study of carbon ion radiotherapy for locally advanced head-and-neck cancer Int J Radiat Oncol Biol Phys 2004 60 2 358 64 10.1016/j.ijrobp.2004.02.067 15380567

[B33] Douglas JG Koh WJ Austin-Seymour M Laramore GE Treatment of salivary gland neoplasms with fast neutron radiotherapy Arch Otolaryngol Head Neck Surg 2003 129 9 944 8 10.1001/archotol.129.9.944 12975266

[B34] Laramore GE The use of neutrons in cancer therapy: a historical perspective through the modern era Semin Oncol 24 672 685 199 9422263

[B35] Maor MH Errington RD Caplan RJ Griffin TW Laramore GE Parker RG Burnison M Stetz J Zink S Davis LW Peters LJ Fast-neutron therapy in advanced head and neck cancer: a collaborative international randomized trial Int J Radiat Oncol Biol Phys 1995 32 599 604 10.1016/0360-3016(94)00595-C 7790244

[B36] Stafford N Waldron J Davies D Walsh-Waring G Smith R Complications following fast neutron therapy for head and neck cancer J Laryngol Otol 1992 106 144 146 10.1017/S0022215100118912 1556488

[B37] Mock U Georg D Bogner J Auberger T Pötter R Treatment planning comparison of conventional, 3D conformal, and intensity-modulated photon (IMRT) and proton therapy for paranasal sinus carcinoma Int J Radiat Oncol Biol Phys 2004 58 147 154 10.1016/S0360-3016(03)01452-4 14697432

[B38] Truong MT Kamat UR Liebsch NJ Curry WT Lin DT Barker FG Loeffler JS Chan AW Proton radiation therapy for primary sphenoid sinus malignancies: treatment outcome and prognostic factors Head Neck 2009 31 1297 1308 10.1002/hed.21092 19536762

[B39] Wu AJ Gomez J Zhung JE Chan K Gomez DR Wolden SL Zelefsky MJ Wolchok JD Carvajal RD Chapman PB Wong RJ Shaha AR Kraus DH Shah JP Lee NY Radiotherapy after resection for head and neck mucosal melanoma Am J Clin Oncol 2010 33 281 285 19823070 10.1097/COC.0b013e3181a879f5

[B40] Zenda S Kawashima M Nishio T Kohno R Nihei K Onozawa M Arahira S Ogino T Proton beam therapy as a nonsurgical approach to mucosal melanoma of the head and neck: a pilot study Int J Radiat Oncol Biol Phys 2010 in press 10.1016/j.ijrobp.2010.04.071 20950948

[B41] Griffin TW Fast neutron radiation therapy Crit Rev Oncol Hematol 1992 13 17 31 10.1016/1040-8428(92)90014-H 1449618

[B42] Weber DC Chan AW Lessell S McIntyre JF Goldberg SI Bussiere MR Fitzek MM Thornton AF DeLaney TF Visual outcome of accelerated fractionates radiation for advanced sinonasal malignancies employing photons/protons Radiother Oncol 2006 81 243 249 10.1016/j.radonc.2006.09.009 17050017

[B43] Hasegawa A Mizoe JE Mizota A Tsujii H Outcomes of visual acuity in carbon ion radiotherapy: analysis of dose.volume histograms and prognostic factors Int J Radiat Oncol Biol Phys 2006 64 396 401 10.1016/j.ijrobp.2005.07.298 16182466

